# Integration of Global Signaling Pathways, cAMP-PKA, MAPK and TOR in the Regulation of *FLO11*

**DOI:** 10.1371/journal.pone.0001663

**Published:** 2008-02-27

**Authors:** P. K. Vinod, Neelanjan Sengupta, P. J. Bhat, K. V. Venkatesh

**Affiliations:** 1 School of Biosciences and Bioengineering, Indian Institute of Technology, Bombay, Powai, Mumbai, India; 2 Department of Chemical Engineering, Indian Institute of Technology, Bombay, Powai, Mumbai, India; University of Nottingham, United Kingdom

## Abstract

The budding yeast, *Saccharomyces cerevisiae*, responds to various environmental cues by invoking specific adaptive mechanisms for their survival. Under nitrogen limitation, *S. cerevisiae* undergoes a dimorphic filamentous transition called pseudohyphae, which helps the cell to forage for nutrients and reach an environment conducive for growth. This transition is governed by a complex network of signaling pathways, namely cAMP-PKA, MAPK and TOR, which controls the transcriptional activation of *FLO11*, a flocculin gene that encodes a cell wall protein. However, little is known about how these pathways co-ordinate to govern the conversion of nutritional availability into gene expression. Here, we have analyzed an integrative network comprised of cAMP-PKA, MAPK and TOR pathways with respect to the availability of nitrogen source using experimental and steady state modeling approach. Our experiments demonstrate that the steady state expression of *FLO11* was bistable over a range of inducing ammonium sulphate concentration based on the preculturing condition. We also show that yeast switched from *FLO11* expression to accumulation of trehalose, a STRE response controlled by a transcriptional activator Msn2/4, with decrease in the inducing concentration to complete starvation. Steady state analysis of the integrative network revealed the relationship between the environment, signaling cascades and the expression of *FLO11*. We demonstrate that the double negative feedback loop in TOR pathway can elicit a bistable response, to differentiate between vegetative growth, filamentous growth and STRE response. Negative feedback on TOR pathway function to restrict the expression of *FLO11* under nitrogen starved condition and also with re-addition of nitrogen to starved cells. In general, we show that these global signaling pathways respond with specific sensitivity to regulate the expression of *FLO11* under nitrogen limitation. The holistic steady state modeling approach of the integrative network revealed how the global signaling pathways could differentiate between multiple phenotypes.

## Introduction

In diploid strains of *Saccharomyces cerevisiae*, transition from a nutrient-rich to a nutrient-limited growth medium is shown to control a switch from budding yeast to filamentous form called pseudohyphae [1]. Filamentous growth is characterized by different cellular processes involving unipolar budding, cell elongation, mitotic delay, agar invasion and cell-cell adhesion [1]–[3]. Diploid cells switch to filamentous growth under nitrogen starvation and is postulated to help the cells in foraging for nutrients [1]. In haploids, this phenomenon is called invasive growth and is shown to occur in rich medium [4]. This dimorphic transition involving different cellular processes is controlled by multiple signaling cascades. Two well characterized pathways involved in filamentous growth are cAMP-protein kinase A (PKA) pathway and mitogen-activated protein (MAP) kinase cascade [4]–[8]. Nutrient responsive TOR signaling cascade is also shown to promote filamentous growth [9], [10]. One of the primary targets of these cascades is *FLO11*, a flocculin gene that is responsible for invasion and pseudohyphae development [8], [11], [12]. Availability of nitrogen source in the medium is shown to control the activation of these signaling pathways, which in turn activates the filamentous growth [7], [13]–[15]. We have previously reported the roles of cAMP-PKA and MAPK pathways with respect to the expression of *FLO11* [16]. However, the regulation of these pathways in response to the availability of nitrogen source was not analyzed. Moreover, cAMP-PKA, MAPK and TOR pathways are global regulators involved in a wide range of functions and therefore, it will be interesting to study how these pathways co-ordinate and signal to achieve a desired phenotype.

**Figure 1:**
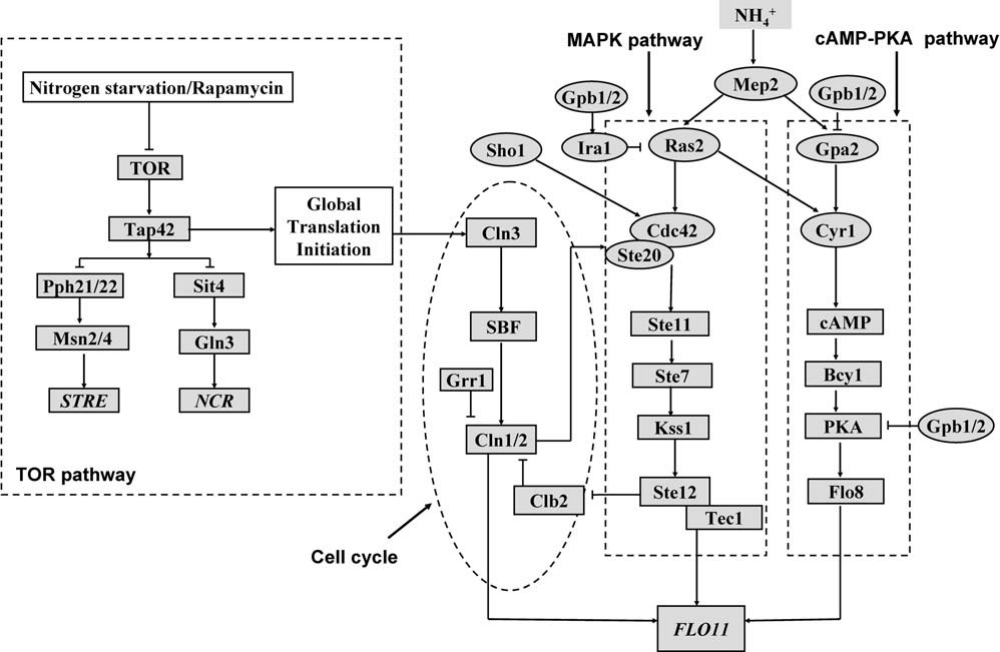
Integrative network of cAMP-PKA, MAPK and TOR pathways involved in the regulation of filamentous growth in Saccharomyces cerevisiae. Mep2 functions in the upstream of cAMP and MAPK pathways as an ammonium sensor. Kelch repeat protein Gpb1/2 antagonizes Gpa2 and PKA of cAMP pathway and stabilizes Ira1, which inactivates Ras2 of MAPK pathway. In cAMP-PKA pathway, Ras2 and Gpa2 activate adenylate cyclase, Cyr1 to synthesis cAMP, which binds to PKA and relieves the inhibition of catalytic subunits Tpk1, Tpk2 and Tpk3. Tpk2 activates the transcriptional activator Flo8 involved in the regulation of *FLO11*. In MAPK pathway, Ras2 and Sho1 activates Cdc42-Ste20 complex, which in turn activates the MAPK cascade Ste11, Ste7 and Kss1 to control the transcriptional activator Ste12-Tec1. Nitrogen starvation or rapamycin treatment is shown to inactivate TOR pathway. Tor kinase phosphorylates Tap42, which complexes with phosphatase Sit4 and Pph21/22. Further, Sit4 and Pph21/22 controls Gln3 mediated NCR genes and Msn2/4 mediated STRE genes, respectively. Tor-activated Tap42 also participate in the global translational initiation. TOR pathway exerts translational control over G1 cyclin Cln3, which in turn controls the synthesis of other G1 cyclin Cln1/2 through transcriptional activator SBF. Cln1/2 is destabilized by Grr1 and is involved in the transcriptional activation of *FLO11*.

cAMP-PKA and MAPK pathways are shown to activate key transcriptional activators Flo8 and Ste12-Tec1 complex, respectively which binds to the distinct region present in the *FLO11* promoter ( see Figure 1) [8], [12]. In cAMP-PKA pathway, Ras2 and Gpa2 activate adenylate cyclase to synthesize cAMP, which in turn relieves the inhibition of Tpks from the regulatory subunit, Bcy1. Tpk2 is shown to activate Flo8 and also remove an inhibitor, Sfl1 [16]–[18]. In MAPK pathway, Ras2 activates Cdc42, which along with Ste20 activate MAPK cascade comprising of Ste11, Ste7 and Kss1 [19]. The MAP kinase, Kss1, activates Ste12-Tec1 to bind to the filamentation response element (FRE) present in the *FLO11* promoter [12], [19]. A high affinity ammonium permease, Mep2 is shown to generate a signal required for the induction of filamentous growth in *S. cerevisiae*, when low concentration of ammonium sulphate is available as nitrogen source [13]. Dominant-active alleles of *GPA2* or *RAS2* or the addition of exogenous cAMP rescued the filamentation defect of Δ*mep2* mutant [13]. Recent work in *Candida albicans* indicated that a dominant *MEP2* allele can overcome the defects either in cAMP-PKA or MAPK, but not both. This study showed that the cytoplasmic c–terminal of Mep2 can activate both cAMP-PKA and MAPK pathways [20]. Earlier study in *S. cerevisiae* had shown that c-terminal domain of Mep2 to be dispensable for filamentous growth [13]. However, recent data from *S. cerevisiae* has suggested that cytoplasmic c-terminal region is required for filamentous signaling and the authors have postulated that Mep2 signals through MAPK pathway. Mep2 is also shown to activate PKA independent of cAMP with readdition of ammonium sulphate to nitrogen starved cells [21]. cAMP independent regulation of PKA is shown to involve kelch repeat protein, Gpb1/2, which directly antagonized the function of PKA [22]. Further, Gpb1/2 negatively regulated the upstream of cAMP-PKA pathway by complexing with Gpa2 and by stabilizing the Ira1/2, a GTPase activating protein of Ras2 (see Figure 1) [22]–[26]. Interestingly, it was observed that mutation in Gpb1/2 demonstrates a hyperfilamentous growth [23]–[25]. Therefore, loss of Gpb1/2 function can be an attractive mode of upstream regulation during conditions favoring filamentous growth. In addition, MAPK pathway also gets an additional input from Sho1-Msb2, a membrane component involved in osmotic signaling [27].

On other hand, signaling pathways sensing the intracellular nitrogen concentration, which gives a measure of availability of extracellular nitrogen, also directly influences the response towards filamentous growth [9], [10]. Tor kinase is shown to be a part of a signal transduction pathway that senses the intracellular nutrients to regulate growth [14], [15], [28]. Intracellular glutamine activated TOR pathway to promote growth, whereas glutamine depletion inhibited TOR pathway to arrest growth. Nitrogen starvation or rapamycin treatment, conditions which inhibited TOR pathway is shown to increase the Gln3 mediated transcription of genes under nitrogen catabolite repression (NCR) and Msn2/4 mediated stress responsive element (STRE) genes [29]–[31]. Nuclear translocation of Gln3p and Msn2/4 is antagonized by TOR pathway under nitrogen rich condition. TOR pathway antagonized the phosphatases Sit4 and Pph21/22 by promoting their association with Tap42 through phosphorylation (see Figure 1) [32]–[34]. Inhibition of TOR pathway is shown to release the phosphatase Sit4 and Pph21/22 from Tap42 and promote the nuclear accumulation of transcriptional activator Gln3 and Msn2/4, respectively [29], [30], [32]–[34]. TOR pathway mediated control of STRE regulated genes include the expression of enzymes involved in the glycogen and trehalose metabolism, which helps to maintain the concentration of these storage carbohydrates during stress [31], [35], [36]. These metabolites get accumulated with the depletion of nitrogen from the medium and were observed concomitantly with the transcriptional activation of genes involved in their metabolism [35]. Further, the regulation of NCR genes by TOR pathway involved Gln3 controlled expression of genes required in the transport and utilization of poor nitrogen source [29].

A role for TOR pathway in regulating filamentous growth has been suggested [9], [10]. Sublethal concentration of rapamycin inhibits filamentous growth in response to nitrogen limitation. TOR pathway downregulates NCR genes, whose product such as Mep2 is actively required for filamentous growth. Loss of TOR pathway is postulated to affect the translation of specific mRNAs required for pseudohyphal growth [9]. This essentially implied that Tor activity is required for filamentous growth. Tor-activated Tap42 is shown to participate in the global translational control under nutrient rich conditions [32], [33], [37]–[39]. Inhibition of Tor activity is shown to result in conditions which inhibit global translation, such as degradation of translation initiation factor eIF4G and phosphorylation of eIF2α [38], [39].

Rapamycin treatment is shown to affect the translation of G1 cyclin *CLN3* mRNA [37]. As a consequence, SBF and MBF mediated expression of G1 cyclins *CLN1* and *CLN2* is downregulated along with increase in the presence of Clb-cyclin kinase inhibitor Sic1, leading to G1 arrest ( see Figure 1) [37]. Nitrogen starvation also resulted in G1 arrest due to the complete loss in the synthesis of G1 cyclins and sustained presence of Sic1 [40]. Interestingly, filamentous growth involves changes in the progression of cell cycle mediated by G1 cyclin Cln1/2/3 and mitotic cyclin Clb2 [3], [41], [42]. The abundance and activity of the G_1_ cyclin Cln1/2 is required for polarization, cell elongation and transcriptional activation of *FLO11*, whereas *Δcln3* mutation enhanced the filamentous growth [41]. Further, a *Δgrr1* mutant, which stabilizes Cln1/2, also enhanced the pseudohyphal growth [41]. G1 cyclin Cln1/2 is shown to modulate Ste20 activity of MAPK pathway for a function independent of transcriptional activation of *FLO11* [43]. On other hand, activation of MAPK is shown to induce a G2/M delay by negatively regulating the mitotic cyclin Clb2 under filamentous growth condition [3], [42]. Another pathway implicated in the regulation of *FLO11* is Sok2 pathway [44]. Inactivation of Sok2 enhanced invasion and filament formation. Transriptional activators Phd1, Ash1 and Swi5 are normally repressed by Sok2 and these activators are shown to regulate the expression of *FLO11*. Evidences suggested that they function in parallel to cAMP-PKA and MAPK pathways [9], [44].

In the current study, we investigate how the variation in the availability of nitrogen source is sensed and transduced by signaling pathways to regulate the expression of *FLO11*. We analyzed an integrative network comprising of cAMP-PKA, MAPK and TOR pathways with respect to varying concentrations of ammonium sulphate using experimental and steady state modeling approach. Our experimental analysis demonstrated that the yeast switched from the expression of *FLO11* to accumulation of trehalose, a storage carbohydrate, with decrease in concentration of ammonium sulphate from limiting to complete starvation. Our analysis also showed that nitrogen starved cells failed to induce the expression of *FLO11* under inducing conditions, thus showing a bistable response with respect to the concentration of ammonium sulphate. We have proposed a model based on the steady state analysis of the signaling pathways to rationalize the experimental observations. Our analysis indicated that the strong double negative feedback loop in the TOR pathway brings about bistable behavior in the expression of *FLO11*. We have also demonstrated that the sensitivity of the specific signaling pathways toward the expression of *FLO11* was different and play a role in defining the resulting phenotype.

## Results

**Figure 2:**
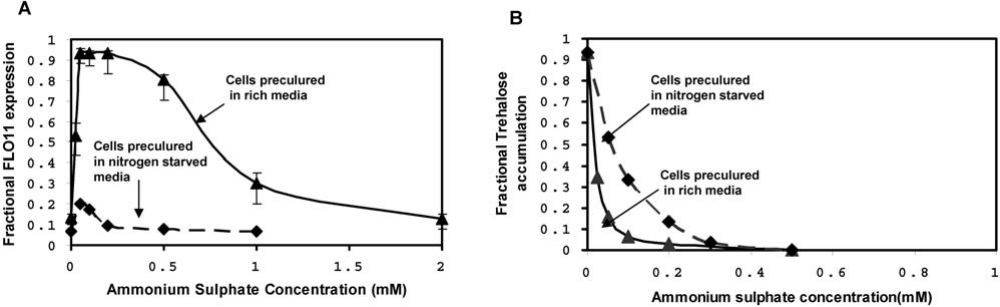
Steady state expression of FLO11 and trehalose accumulation at different concentrations of ammonium sulphate. (a) Fractional expression of *FLO11* with respect to the concentration of ammonium sulphate, when cells are precultured in nitrogen rich medium (solid line) and nitrogen starved medium (dashed line). (b) Fractional accumulation of trehalose with respect to the concentration of ammonium sulphate, when cells are precultured in nitrogen rich medium (solid media) and nitrogen starved medium (dashed line).

Steady state expression of *FLO11* was measured at different limiting concentrations of ammonium sulphate, when the cells were precultured in the medium containing excess of ammonium sulphate. It should be noted that ammonium sulphate was the sole nitrogen source. The expression of *FLO11* increased as the steady state concentration of ammonium sulphate decreased below 1 mM and increased to a maximum (solid line in Figure 2a). Further, the expression of *FLO11* decreased steeply for the concentration of ammonium sulphate below 25 µM and reached a minimum value. Thus, expression of *FLO11* was observed within the range of 25 µM-1 mM of ammonium sulphate. The intracellular trehalose concentration (the storage carbohydrate) was also measured under these conditions. Trehalose concentration increased below 25 µM concentration of ammonium sulphate and reached a maximum under complete starvation condition (solid line in Figure 2b). Experiments were also conducted at different steady state concentration of ammonium sulphate with cells precultured in a medium lacking ammonium sulphate. In this case, with increase in the concentration of nitrogen, the expression of *FLO11* did not get activated and remained switched off under all conditions (dotted line in Figure 2a). This indicated that the expression of *FLO11* was restricted when nitrogen starved cells were transferred to the medium containing inducing concentrations of ammonium sulphate. However, the intracellular trehalose concentration decreased with increase in the steady state concentration of ammonium sulphate and was zero at ammonium sulphate concentration exceeding 100 µM (dotted line in Figure 2b). The profile of trehalose accumulation was different for the two preculturing conditions in response to the varying concentrations of nitrogen (Figure 2b). Thus, the expression of *FLO11* and accumulation of trehalose demonstrated a bistable response over a range of ammonium sulphate concentration, which depended on the preculturing condition of the cells.

### Steady state model to quantify the expression of *FLO11* with respect to ammonium sulfate concentration

**Figure 3:**
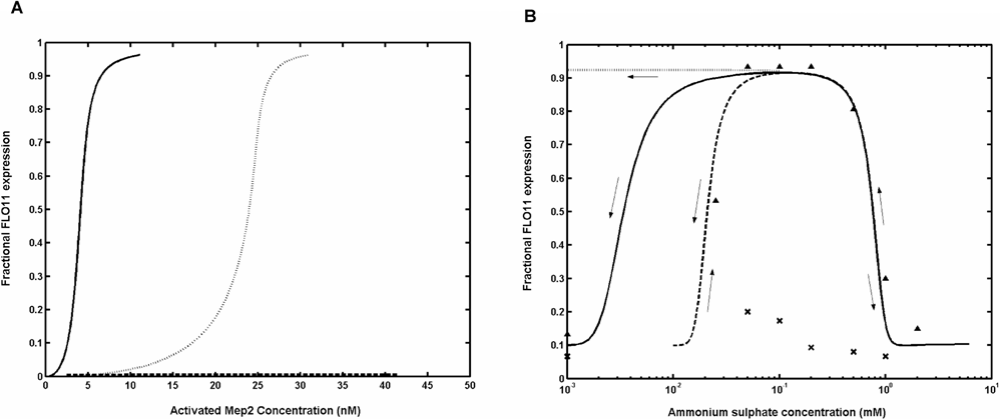
Fractional activation of FLO11 by cAMP-PKA and MAPK pathways. (a) Dose response curve for the expression of *FLO11* with respect to the upstream component, Mep2 under wild type (solid line), *Δgpa2* mutant (dotted line) and *Δgpa2ras2* mutant (dashed line) conditions. (b) Fractional expression of *FLO11* with respect to the concentration of ammonium sulphate obtained using the network comprising of cAMP-PKA and MAPK pathways. Dose response was evaluated for K = 2 µM (Km for ammonium) (solid line), K = 16 µM (dashed line) and without the first term (dotted line) in Equation 4. The experimental values for the expression of *FLO11* with cells precultured in nitrogen rich medium (▴) and nitrogen starved medium(x) are indicated. It can be noted that the simulation matched the data obtained from preculturing in nitrogen rich medium (▴), while not able to simulate the data for preculturing in nitrogen starved medium (x).

In our previous study, the signaling network involving cAMP-PKA and MAPK pathways were quantified through a steady state model with respect to upstream components adenylate cyclase and Cdc42 [16]. However, Mep2, which senses the concentration of ammonium sulfate, functions upstream of cAMP-PKA and MAPK pathways was not included in the previous model. Therefore, we began our analysis by analyzing these two pathways with respect to Mep2. The upstream regulation of cAMP-PKA and MAPK pathways by Mep2 were integrated into our existing steady state model of these pathways (Figure S1). Simulations were carried out by varying the fractional activation of cAMP-PKA and MAPK pathways to get the dose response curves with respect to Mep2. Figure 3a (solid line) shows the predicted response curve for the expression of *FLO11*, which demonstrated a highly sensitive response, with a Hills coefficient of 4 and K_0.5_ of 4nM with respect to activated concentration of Mep2. It was interesting to note that the sensitivity as Hills coefficient was same as obtained with respect to adenylate cyclase of cAMP-PKA pathway (see Equation 3). We have previously attributed the high sensitivity observed in the expression of *FLO11* to inhibitor ultrasenitivity [16]. We also obtained the dose response curve for an in-silico mutant lacking Gpa2 in the cAMP-PKA pathway (dotted line). In this case, the expression of *FLO11* was shifted to the right indicating a higher requirement of activated Mep2. The profile demonstrated a highly sensitive response with a Hills coefficient of 8 and K_0.5_ of 24 nM. A six fold higher amount of activated Mep2 was required as compared to the wild type to overcome the defects of Gpa2 mutation as observed with the dominant allele of *MEP2* [20]. This increase in the sensitivity was mainly due to the hyperactivation of both cAMP-PKA and MAPK pathways brought about by the positive feedback of Cdc25 on Ras2 activation (Figure S1). A dose response curve for a mutant lacking both Gpa2 and Ras2 resulted in complete loss of *FLO11* expression irrespective of fold change in the activation of Mep2 (dashed line). This was in accordance with genetic studies which demonstrated that Mep2 signals through both cAMP and MAPK pathways [20].

The concentration of activated Mep2 was correlated to the extracellular concentration of ammonium sulphate since the expression of *FLO11* was experimentally evaluated to changes in the concentration of ammonium sulphate. A Hill type equation including inhibition by ammonium sulphate was used to relate the concentration of ammonium sulphate to Mep2 (see Equation 4). The values of nH8, nH9 and K_9_ were obtained by fitting the experimental data, while the value of K was obtained from the literature [13]. The value of K reflected the limitation in Mep2 activation through ammonium transport. The comparison of the predicted dose response curve with the experimental data is shown in Figure 3b (solid line). It can be seen that when cells from a medium containing higher concentration of nitrogen was introduced into the medium containing limiting concentration of nitrogen, the expression of *FLO11* increased with Hills coefficient of 2 and remained activated in the range of 6 µM–300 µM. However, the experiments indicated a range of 25 µM–300 µM in which *FLO11* was fully expressed. The predicted response matched the concentration of ammonium sulphate at which the expression of *FLO11* was switched on, but did not match the concentration required for switching off. A steady state response curve was obtained by neglecting the first term of Equation 4 and it was observed that the expression of *FLO11* did not drop at low concentration of ammonium sulphate (see dotted line in Figure 3b). Therefore, the value of ‘K’ was increased from the reported value of 2 µM (Km for ammonium) to 16 µ (8 fold increase) to match the experimental profile (see dashed line in Figure 3b). This analysis indicated that the drop in the expression of *FLO11* depended on the first term of Equation 4, which essentially functions as a mechanism to restrict the expression of *FLO11* under starvation. However, it is not clear regarding the molecular mechanism involved in decreasing the expression of *FLO11*. We also simulated the condition wherein cells precultured on nitrogen starved medium were introduced into medium containing various limiting concentrations of ammonium sulphate. Simulation results showed that the expression of *FLO11* was reversible and did not depend upon the preculturing condition (see Figure 3b). This indicated that the signaling structure present in the network comprising of cAMP-PKA and MAPK pathways do not contribute to the bistable behavior as observed in our experiments.

The other relevant pathway involved in regulating the expression of *FLO11* depending on the availability of nitrogen was TOR pathway. Therefore, we also modeled the TOR pathway at steady state to evaluate the influence on the expression of *FLO11*. The Tor activity is shown to be higher under nitrogen rich and lower under nitrogen starvation condition [29], [30]. However, the variation of TOR pathway with the availability of nitrogen source has not been analyzed before. Further, loss of Tor activity resulted in a complete loss of G1 cyclins by translational repression and degradation, which led to G1 arrest [37]. In contrast, filamentous growth observed under nitrogen limiting condition required the activity of G1 cyclins Cln1/2 and the removal of inhibitory function of Cln3 [41]. Moreover, Tor activity was also needed for the translation of specific mRNAs involved in the filamentous growth indicating a positive role in this phenotype [9]. This suggested that filamentous growth might still require a signal confirming the limited presence of nitrogen only through partial loss of Tor activity. In order to understand such a variation in Tor activity with availability of nitrogen source, we simulated the possible solution space under which Tor kinase can function to regulate the expression of *FLO11*. We assumed a Hill type dependence between fractional Tor activation and ammonium sulphate concentration as given by Equation 5. We performed a parametric sensitivity analysis to evaluate the feasible values for nH and K_0.5_ in Equation 5.

**Figure 4:**
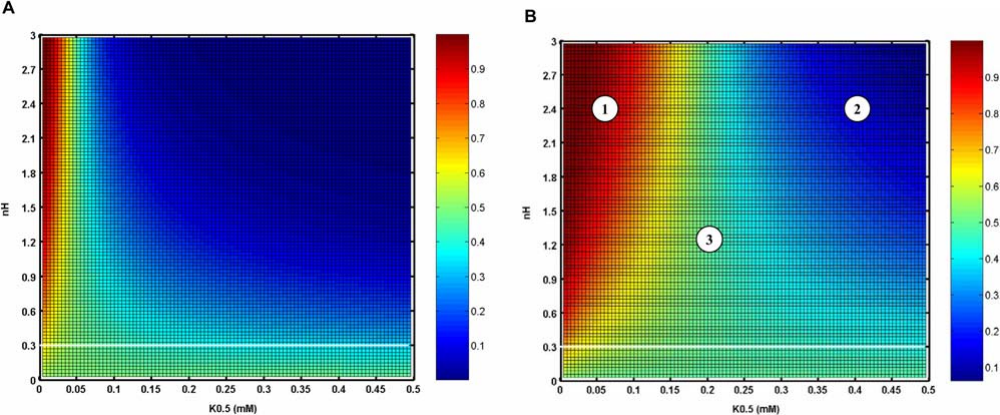
Sensitivity analysis for the fractional activation of Tor with respect to the concentration of ammonium sulphate. Surface plot with varying values of Hills coefficient, nH4 and half saturation constant, K_4 _(Equation 5) at ammonium sulfate concentration of (a) 50 µM and (b) 200 µM. The fractional activation of Tor is shown as colour bar. Region 1 represents the activity of Tor greater than 80% (red), region 2 represents the activity of Tor less than 20%( blue) and region 3 represents the activity of Tor between 30-70%. The region over which Tor operates to regulate the filamentous growth is for nH4 value less than 0.3(below the white line).

In our experiments, the expression of *FLO11* was maximum over a wide concentration range of ammonium sulphate, i.e 25 µM–300 µM. Since certain level of Tor activity was essential for the expression of *FLO11*, the activity of Tor should be maintained in this range. This implied that the variation in the activity of Tor on ammonium sulphate concentration between 25 µM–300 µM should not be drastic. Further, the activity of Tor should be high under normal growth promoting condition, while should be low under complete starvation [29], [30]. Hence, the parameter values should be such that it yields a partial Tor activity in the range where *FLO11* was completely expressed. Based on the above criteria, we simulated a surface plot for the ammonium sulphate concentration of 50 µM and 200 µM at varying nH and K_0.5_ values (Figure 4a and 4b). For both 50 µM and 200 µM concentration of ammonium sulphate, there existed three distinct regions comprising of fractional activity of Tor greater than 80% (region-1, red zone), lesser than 20% (region-2, blue zone) and in between 30-70% (region-3 in Figure 4b). A comparison between these two surface plots yielded a common region of nH and K_0.5_ values, where the difference in the activity of Tor was minimum and also not a part of a region with high or low Tor activity. We observed that such a common region existed for nH value lesser than 0.3 and K_0.5_ greater than 0.01mM, where the difference in Tor activity was less than 10% (region below the white line in Figure 4). Such a region represented the possible range of nH and K_0.5_ values over which Tor kinase can function to regulate the filamentous growth. This essentially indicated that Tor activity was subsensitive to the concentration of ammonium sulphate.

**Figure 5:**
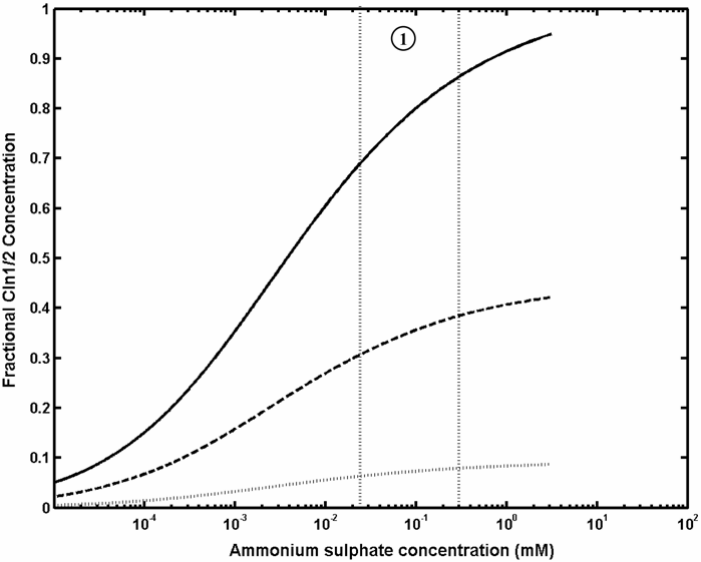
Cln1/2 synthesis with respect to the concentration of ammonium sulphate. The dose response for the fractional concentration of Cln1/2 at 90%(dotted line), 50%(dashed line) and 10%(solid line) concentration of Clb2. The experimental observation of *FLO11* expression over a concentration range of ammonium sulphate is indicated using dotted lines (marked as region 1).

Under these conditions, we simulated the dose response for Tor kinase mediated control of G1 cyclin, Cln1/2 synthesis with respect to the concentration of ammonium sulphate (see Figure 1 and Figure S2). The dose response was evaluated for fixed concentration of mitotic cyclin Clb2. Figure 5 shows the predicted response curve for the fractional Cln1/2 concentration, which varied depending upon the concentration of Clb2. Dotted, dashed and solid lines indicated the response curve for 90%, 50% and 10% of the total concentration of Clb2, respectively. For a minimum concentration of Clb2 (less than 10%), the dose response shows a subsensitive response with a Hills coefficient of 0.3 and the fractional Cln1/2 concentration was at a higher steady state for higher concentration of ammonium sulfate (solid line). However, with increase in the concentration of Clb2, the fractional Cln1/2 concentration decreased to a lower steady state value (dashed and dotted line). Furthermore, the fractional Cln1/2 concentration was low irrespective of Clb2 concentration at lower concentration of ammonium sulphate due to the limitation in the activity of Tor. This analysis indicated that the concentration of Cln1/2 strongly depended on the activity of Tor and the concentration of Clb2. Moreover, in the concentration range wherein filamentous growth was induced, Cln1 concentration was at a higher steady state only if the concentration of Clb2 was at a minimum (solid line in region-1). Studies have shown that the activity of mitotic cyclin Clb2 decreases during filamentous growth to induce mitotic delay [3], [42]. Therefore, such a decrease in mitotic cyclin activity can also account for the high activity of G1 cyclin Cln1/2 observed during the filamentous growth. Under higher concentration of ammonium sulphate, the concentration of Cln1/2 can switch between higher and lower steady state depending upon the concentration of Clb2, which was indicative of the budding cell cycle progression.

**Figure 6:**
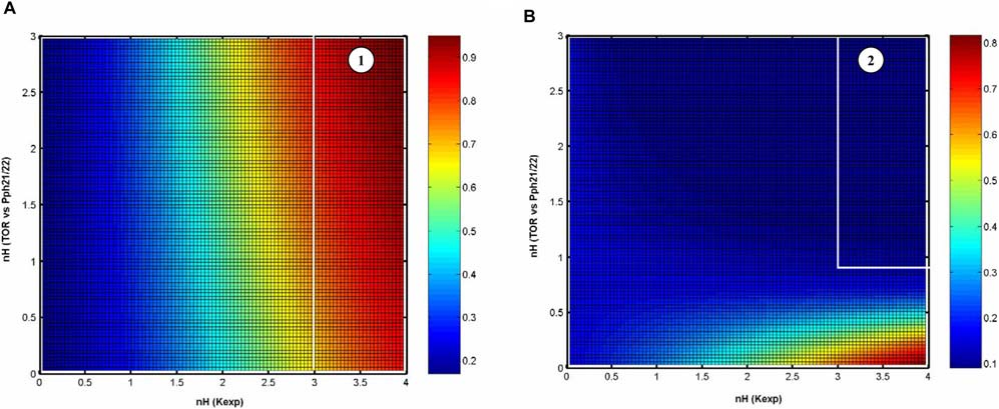
Sensitivity analysis for the fractional nuclear accumulation of Msn2/4. The surface plot with varying values of Hills coefficient for Tor regulation of Pph21/22 (nH13 in Equation 7) and Hills coefficient for Pph21/22 regulation of Msn2/4 export (nH11 in Equation 6) at (a) 10% and (b) 90% Tor activity. The fractional nuclear accumulation of Msn2/4 is shown as a colour bar. The region 1 represents the fractional nuclear accumulation of Msn2/4 greater than 70% (red) and region 2 represents the fractional nuclear accumulation of Msn2/4 less than 10% (blue).

TOR pathway also regulated the Msn2/4 mediated transcription of STRE genes through control of Msn2/4 translocation (see Figure 1 and Figure S2). TOR pathway controlled Msn2/4 independent of cAMP-PKA pathway since PKA dependent phosphorylation of Msn2-NLS was unaffected under nitrogen starvation [45]. To study the effect of TOR pathway on Msn2/4 translocation, input from the cAMP-PKA pathway was held constant (i.e. the term Tpk in Equation 6). This resulted in a higher export rate from the nucleus with 90% of Msn2/4 accumulating in the cytoplasm. The nuclear accumulation of Msn2/4 occurred with loss in the activity of Tor under nitrogen starvation. Tor kinase mediated this effect through negative regulation of the phosphatase Pph21/22 as given by Equation 7. We have analyzed the sensitivity with which TOR pathway regulated the phosphatase Pph21/22 and thereby the export rate of Msn2/4. The sensitivity for the negative regulation of phosphatase Pph21/22 by Tor kinase is given by Hills coefficient ‘nH13’ in Equation 7. Similarly, the sensitivity for the regulation of export rate by Pph21/22 is given by Hills coefficient ‘nH11’ in Equation 6. Figure 6 shows the surface plot for the fractional nuclear accumulation of Msn2/4 at 10% and 90% activity of Tor with varying values of ‘nH11’ and ‘nH13’. The values of K_0.5_ were fixed based on the assumption that maximum nuclear accumulation of 90% occurred when the activity of Tor was minimum (assumed to be 10%). Figure 6a indicated that for a minimum Tor activity of 10%, the nuclear accumulation of Msn2/4 varied from 70–100% for nH11 value between 3–4 and nH13 value between 0–3 (marked as region-1). Further, in this range, the nuclear accumulation of Msn2/4 should be less than 10% for a high Tor activity of 90%. The plot clearly showed that with 90% Tor activity, the nuclear accumulation of Msn2/4 was less than 10% for the values of nH11 and nH13 between 3–4 and 0.8–3, respectively (region-2, Figure 6b). In our experiments, accumulation of trehalose, which was related to STRE activity regulated by Msn2/4, started to increase gradually as the concentration was decreased below 25 µM with a Hills coefficient of 0.5 (Figure 2b). Thus, the fractional accumulation of trehalose demonstrated a subsenstive response, which suggested that nH13 takes a value between 0.9 and 1.2. We therefore concluded that TOR regulation of phosphatase Pph21/22 was less sensitive and fixed a value of 3 and 1.1 for nH11 and nH13, respectively.

Further, we simulated the dose response curve by incorporating the TOR pathway into the network comprising of cAMP-PKA and MAPK pathways to match our experimental observation with regard to the expression of *FLO11*. We characterized the network behavior as multiplicative product of three functionalities involving cAMP-PKA, MAPK and TOR pathways to evaluate the fractional expression of *FLO11*.
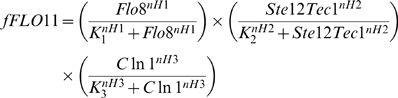
(1)


Equation 1 included the transcriptional activation of *FLO11* by Flo8, Ste12-Tec1 and Cln1 controlled by cAMP-PKA, MAPK and TOR pathways, respectively. In our previous study, we have shown that the expression of *FLO11* was highly sensitive to cAMP-PKA pathway (Hills coefficient nH = 4 and K_0.5_ = 67 nM) with respect to the concentration of Flo8. Similarly, the expression *FLO11* was shown to be subsentive to MAPK pathway (Hills coefficient, nH = 0.8 and K_0.5_ = 10 nM) with respect to the concentration of Ste12-Tec1 [16]. However, the sensitivity with which Cln1 regulated the expression of *FLO11* remained to be evaluated. Therefore, we simulated the network with different values of nH3 and K_3_ in Equation 1 to get the best fit with the experimental data. We observed that for nH3 value greater than 3 and K_3_ value less than 3 nM, the dose response obtained were similar to the response curve shown in Figure 3b (solid line). For K_3_ values higher than 3nM, the fractional expression of *FLO11* decreased below 90% (result not shown). This analysis indicated that with the inclusion of TOR pathway, the expression of *FLO11* with respect to changes in the concentration of ammonium sulphate was unchanged (i.e. reversible). Thus, TOR pathway essentially integrated the STRE response controlled by Msn2/4 without any effect on the expression of *FLO11*. This was in sharp contrast with experimental observations which had demonstrated a bistable behavior with both expression of *FLO11* and accumulation of trehalose based on the preculturing conditions. However, these phenotypes are strongly linked to the activity of Tor and therefore, any variation in the activity of Tor with preculturing conditions might bring about the difference in the expression of *FLO11*. For example, nitrogen starved cells which are shown to be depleted of G1 cyclins needed to synthesize the degraded cyclins when transferred to a media containing inducing concentration of filamentous growth. However, the cells grown on nitrogen rich media had higher activity of G1 cyclins. The experiments carried out with cells precultured in nitrogen rich and starved medium had this difference with regard to the protein level of G1 cyclins. We hypothesized this difference in cyclin concentration might bring about a differential expression in *FLO11*, as G1 cyclin Cln1/2 was shown to be a transcriptional activator of *FLO11* [41]. This difference resulted only if we assumed that there was a difference in the activity of Tor under these two conditions. TOR pathway mediated negative regulation of phosphatase Pph21/22 involved phosphorylation of Tap42, which formed a complex with Pph21/22 to downregulate its activity [32], [33]. In turn, Pph21/22 was shown to dephosphoryate Tap42, thereby function negatively with respect to activity of Tor. These results indicated the presence of a negative feedback on the activity of Tor. Hence, we postulated that complete activation of Pph21/22 under nitrogen starvation condition might antagonize the activity of Tor with re-addition of nitrogen source. Under these circumstances, higher concentration of nitrogen source was required to overcome the antagonism. Thus, Tor activity can be quantified as,
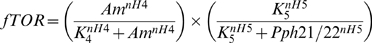
(2)


**Figure 7:**
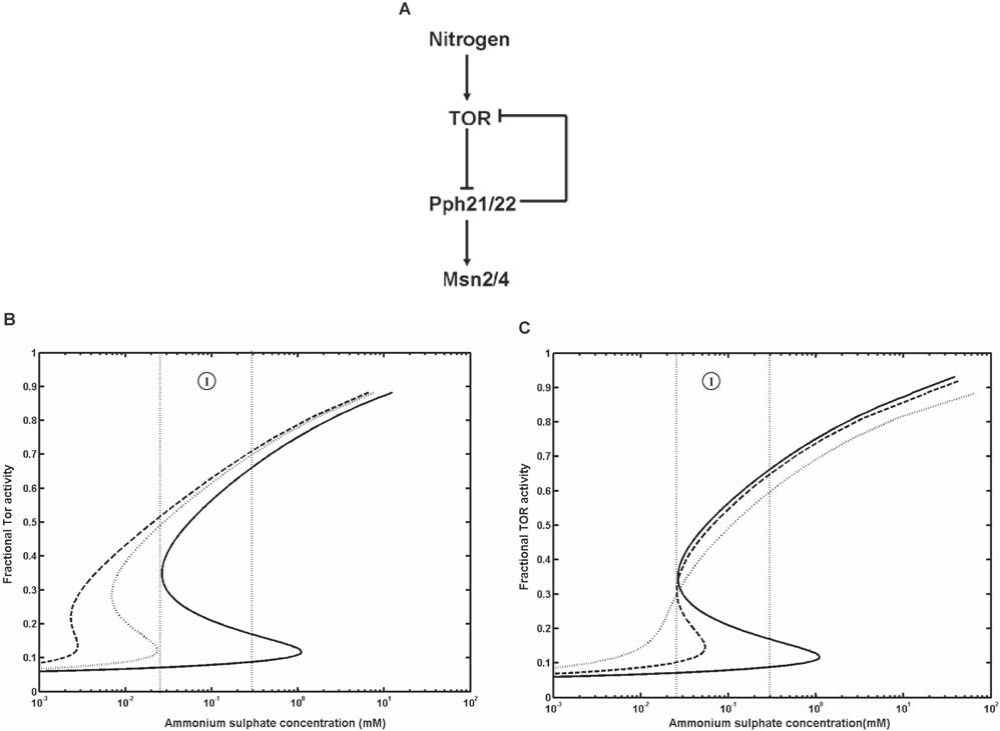
Bistable response of the TOR pathway with respect to the concentration of ammonium sulphate. (a) A simplified diagram showing a negative feedback on Tor, which constitutes a double negative feedback loop in the TOR pathway. The effect of negative feedback on the bistable response for (b) K_5_ = 5 nM (solid line), 6 nM (dotted line) and 7nM (dashed line) and for (c) nH5 = 3 (solid line), 2.5 (dashed line) and 2(dotted line) in Equation 2. The experimental observation of *FLO11* expression over a concentration range of ammonium sulphate is indicated using dotted lines (marked as region 1).

Such a negative feedback on the activity of Tor completed the double negative feedback loop in the TOR pathway (Figure 7a). Tor protein inhibited or repressed the phosphatase Pph21/22, and in turn Pph21/22 inhibited or repressed Tor, which constituted a double negative feedback loop. Simulation of the fractional activity of Tor under these conditions shows a bistable response with respect to the concentration of ammonium sulphate (Figure 7b and Figure 7c). The strength of the negative feedback was varied to study its effect on the dose response. With decrease in K_5_ (Figure 7b) or increase in nH5 (Figure 7c) in Equation 2, the dose response can be made to be bistable over a wide range of ammonium sulphate concentration.

**Figure 8:**
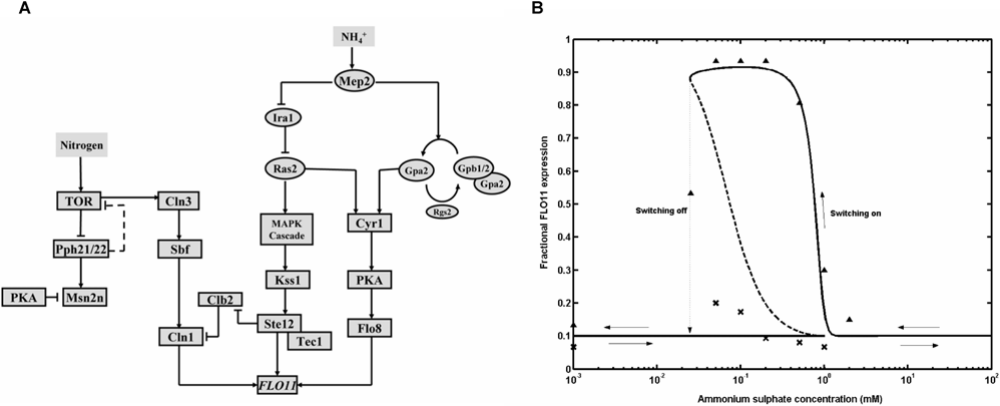
Simulated dose response for a network comprising of cAMP-PKA, MAPK and TOR pathways with negative feedback on Tor included. (a) A simplified network comprising of different components of cAMP-PKA, MAPK and TOR pathways considered for the simulation are shown (b) Bistable response of *FLO11* expression over an inducing concentration range of ammonium sulfate. *FLO11* expression exists in two stable states, i.e. either high or low based on the preculturing condition. The intermediate state is unstable, which is shown as dashed line. The experimental values for the expression of *FLO11* with cells precultured in nitrogen rich medium (▴) and nitrogen starved medium (x) are indicated.

We also simulated the dose response for the entire network comprising of cAMP-PKA, MAPK and TOR pathways including the negative feedback on the activity of Tor (see Figure 8a). The network behavior was evaluated in terms of fractional expression of *FLO11* using Equation 1. The simulated dose response matched well with the experimental values for a Hills coefficient of 4 and K_0.5_ of 2.5 nM with respect to the concentration of Cln1 (see Figure 8b). This indicated that the expression of *FLO11* was highly sensitive to the concentration of Cln1, which in-turn depended on the fractional activation of TOR pathway. Moreover, the simulated dose response for the expression of *FLO11* shows a bistable behavior over the inducing concentration range of ammonium sulphate. Depending upon the direction of the simulation, the expression of *FLO11* switched between stable steady states, with higher state favored only under downshift from high to inducing concentration of ammonium sulphate. Similarly, the expression of *FLO11* failed to respond under upshift from starvation to inducing concentration of ammonium sulphate, which resulted in an irreversible response (see Figure 8b). However, the expression of *FLO11* can be achieved provided the cells are again precultured in a medium containing higher concentration of nitrogen and later exposed to the inducing concentration of nitrogen. This indicated that cells are responding to the downshift in the nitrogen availability, which was required to trigger the expression of *FLO11*. The observed bistability in expression of *FLO11* can be directly related to the bistable behavior of the TOR pathway. Under vegetative growth conditions (i.e., concentration of ammonium sulphate greater than or equal to 1mM), the activity of Tor was high and demonstrated a subsensitive response with respect to the concentration of ammonium sulphate (see Figure S3). However, in the region between 25 µM to 1mM concentration of ammonium sulfate, the activity of Tor demonstrated a bistable response, which remained subsensitive to the lowering of nitrogen concentration, but was switched off in response to the increasing nitrogen concentration. For concentration of ammonium sulphate below 25 µM, the activity of Tor remained switched off, which helped the cells in activating the STRE responses under nitrogen starvation. Thus, the activity of Tor shifted from a subsensitive response to a bistable response when cells are precultured in nitrogen rich condition and exposed to varying concentration of nitrogen. However, when cells are precultured in nitrogen starved medium, the activity of Tor switched on only after remaining switched off in both starvation and filamentous growth regions (i.e., beyond 1mM of ammonium sulphate concentration). As expected when the feedback of Pph21/22 on Tor was removed, the bistability vanished and only a subsensitive response was observed (Figure S3).

## Discussion

In our current study, we have analyzed the regulation of filamentous growth in relation to the availability of nitrogen in the medium. We have quantified the expression of *FLO11* at different steady state concentration of ammonium sulphate. The observed systemic behavior was co-related to the structure of the signaling network with the help of a steady state model. In our experiments using liquid medium, we have demonstrated that the cells precultured in nitrogen rich medium were able to switch on the expression of *FLO11* when transferred to media containing limiting concentration of ammonium sulphate. However, with the concentration of ammonium sulphate decreased towards starvation, the expression of *FLO11* was switched off and the accumulation of trehalose increased to a maximum. Further, cells in the liquid media responded by inducing the filamentous growth involving the expression of *FLO11*, however without the formation of filament. The expression of *FLO11* was required for the formation of filament in solid media, which helped the cells to forage for nutrients and reach the environment conducive for growth [1]. This difference observed with liquid media can result from the fact that the cells are responding to an uniform concentration of ammonium sulphate, which is sufficient for the expression *FLO11*, but not for the formation of filaments.

We have analyzed the switching on and switching off function of the expression of *FLO11* by considering a network comprising of cAMP-PKA, MAPK and TOR pathways, whose involvement in filamentous growth is well studied. Steady state model developed based on these pathways indicated that the expression of *FLO11* switched on by the activation of both cAMP-PKA and MAPK pathways and with partial loss of Tor activity under inducing concentration of ammonium sulphate. The expression of *FLO11* was highly sensitive to the concentrations of Flo8 (cAMP-PKA pathway) and Cln1/2(TOR pathway), whereas subsensitive to the concentration of Ste12-Tec1 (MAPK pathway) (see Equation 1). The repression of *FLO11* expression under severe limitation in the concentration of ammonium sulphate primarily depended on the Tor activity. The activity of Tor decreased from partial loss during the expression of *FLO11* (i.e. between 25 µM to 300 µM concentration of ammonium sulphate) to complete repression below 25 µM concentration of ammonium sulphate. This was evident from the fact that the decrease in Tor activity increased the STRE activity controlled by Msn2/4 resulting in the accumulation of trehalose. Under complete starvation condition, both cAMP-PKA and MAPK pathways do not get activated due to the absence of ammonium transport through Mep2, which along with complete loss of Tor activity contributed towards restricting the expression of *FLO11*. Moreover, we also demonstrated that cAMP-PKA and MAPK pathways can remain inactive with decrease in the concentration of ammonium sulfate towards starvation (i.e. below 25 µM) to restrict the expression of *FLO11*, provided a mechanism exist to antagonize its activation (see Figure 3b). Increase in STRE activity due to loss in the activity of Tor might antagonize the activation of cAMP-PKA and MAPK pathways. However, we rule out this possibility since the conditions which inhibited the activity of Tor does not affect the phosphorylation state of Msn2-NLS controlled by PKA of cAMP pathway [45]. Therefore, loss of TOR activity does not spill into cAMP-PKA pathway indicating that the TOR pathway function parallel to cAMP-PKA pathway. Evidences showed that cAMP-PKA, MAPK and TOR pathways function in parallel to regulate the expression of *FLO11*, where loss of function in either of these pathways was sufficient to restrict the expression of *FLO11* [8], [9], [44]. Therefore, in the regulatory network of filamentous growth, multiple signaling pathways function parallely to integrate the signals onto the *FLO11* promoter and thereby control its expression.

Our analysis also quantified the difference between nitrogen starvation (less than 25 µM concentration of ammonium sulphate) and nitrogen limitation (25 µM-300 µM concentration of ammonium sulphate). The signaling events during these phases differ distinctly resulting in cells preferring the accumulation of trehalose to filamentous growth under nitrogen starvation, while preferring filamentous growth under condition of nitrogen limitation. Our analysis indicated that TOR pathway was able to distinguish between these two states to trigger appropriate response in order to survive under nutritional stress. Such difference in the intensity of stress is shown to trigger different response even in the case of other stress responses such as in osmotic stress, where cells survived severe osmotic stress by accumulating trehalose and moderate osmotic stress through glycerol accumulation [46], [47].

Further, our experiments with cells precultured on nitrogen starved media failed to induce the expression of *FLO11* when transferred to media containing inducing concentration of ammonium sulphate. This suggested that the expression of *FLO11* was bistable over inducing concentration range of ammonium sulphate and can be either high or low depending on the preculturing state. Such an observation can be directly correlated to the presence of a positive feedback or double negative feedback operating within the filamentous signaling network, which is known to exhibit bistable response [48], [49]. Moreover, the bistable system can become irreversible in the presence of a strong feedback. A strong positive feedback loop activates a phenotypic response in the presence of stimulus, but also prevents the response from turning off on removal of stimulus. In the case of double negative feedback loop, the phenotypic response is activated in the presence of stimulus and becomes inactivated on removal of stimulus, but also prevents the activation on re-introducing the stimulus. We observed that such a behavior brought about by double negative feedback loop resembled the response observed in our experiment with regard to the expression of *FLO11*.

In the network comprising of cAMP-PKA and MAPK pathways, we did not observe the presence of double negative feedback loop which can contribute towards a bistable response. However, in TOR pathway, the phosphatase Pph21/22 functioned reciprocally to the activity of Tor yielding a double negative feedback loop in the network (see Figure 8). Such a negative feedback on the activity of Tor antagonized its role in filamentous growth and made the system irreversible. Interestingly, evidence suggested that the increase in Msn2/4 activity can suppress the filamentous growth [50]. Under nitrogen starvation condition, increase in Msn2/4 activity depended on the activation of phosphatase Pph21/22 and therefore can function to antagonize filamentous growth either directly or through a gene product activated by the transcriptional activator Msn2/4. This indicated that under inducing condition of filamentous growth, the cells have differed STRE responses to accommodate the adaptive response of filamentous growth by a mechanism which prevented or antagonized the Msn2/4 activity. Strains which are incapable of antagonizing the Msn2/4 activity under filamentous growth conditions have shown to lack the adaptive response of filamentous growth [50]. Such a negative feedback regulation can maintain the off state of filamentous growth on addition of ammonium sulphate to nitrogen starved cells. In general, our analysis quantified the strength of the negative feedback, which made the system irreversible over a concentration range of ammonium sulphate. This interaction might be necessary as cells preferred degradation of reserves to specific adaptive response, while recovering from nitrogen starvation.

Mep2 and Tor kinase take part in sensing and signaling the availability of nitrogen source. Our analysis indicated that Mep2 signaling function was sensitive to the concentration of ammonium sulphate, whereas the activity of Tor was insensitive. Such difference in the sensitivity was necessary to achieve the desired properties of a phenotype. Furthermore, the difference in sensitivity also reflected their global role in signaling towards other phenotypes. For example, TOR pathway is shown to be a central controller of cell growth and variation in its activity strongly affected the growth [14], [15], [28]. Under such circumstances, if Tor activity was sensitive to nutrients, then for a small change in the concentration of nutrients, the activity of Tor would have decreased dramatically to inhibit growth, which might be undesirable. Therefore, the activity of Tor was subsensitive to the nutritional variation, which made the Tor mediated control of G1 cyclins and STRE regulated genes also less sensitive. This helped the cells to grow albeit slowly under nutritional limitation. Moreover, Tor kinase was able to perform multiple tasks such as control of vegetative growth through a subsenitive response, filamentous growth through a bistable response and starvation response by remaining switched off. In case of Mep2, since it was involved in the transport of ammonium sulphate under both higher and lower concentrations, its function towards signaling was restricted only under lower concentrations. Hence, cells switched to an adaptive response for the survival when the nutrient availability reduced below the threshold requirement for vegetative growth. This was possible only due to the sensitive response of Mep2 signaling to the concentration of ammonium sulphate.

The steady state analysis in tandem with experiments demonstrated how a complex signaling network interlinks various pathways to respond to changes in the nutritional status. Specific to the expression of *FLO11*, three pathways cAMP-PKA, MAPK and TOR operated parallely to decide the appropriate phenotypic response based on the degree of nutritional availability. It should be noted that these pathways have global role in regulating multiple phenotypes and therefore, variation in the sensitivities of these pathways toward each phenotype might help the cells to achieve the desired phenotype. We have demonstrated how these signaling pathways with specific sensitivity towards expression of *FLO11* are integrated to respond under nitrogen starvation. It will be interesting to study the sensitivity with which these global players regulate other phenotypic responses.

## Materials and Methods

The general methodology that was used to characterize the steady state behavior of the signaling network involved in regulating a phenotypic response is shown as a flowsheet in Figure S4. The first step involved the experimental quantification of phenotypic response at steady state in terms of signal input to output response of the network. We have quantified the expression of *FLO11* as an output response with respect to steady state concentration of ammonium sulphate, which acted as an input. The observed systemic behavior was analyzed using the steady state model of the integrative network of signaling pathways as available in the literature. The experimental observations were compared to the results obtained from the steady state model. Based on the comparison of the results to that obtained through experiments, possible hypotheses can be evoked to rationalize the experimental observations. The simulated input-output dose response curves indicated the operation and design of the signaling structure necessary to elicit the observed systemic properties.

### Yeast Strain and Growth Conditions

The strain (MLY61a/α) used in this study was a derivative of the *S. cerevisiae* Σ*1278b* background [13]. A *FLO11*-lacz reporter plasmid was used to quantify the expression of *FLO11* [8]. Yeast transformation was carried out using lithium acetate method [51]. The analysis was confined to the selective media so that the strains retained the plasmid. The cells were pre-grown to mid-log phase in liquid synthetic minimal medium (SD) at 30°C, washed twice with 2% glucose and inoculated into SLAD (2% glucose, 0.17% YNB without ammonium sulfate and amino acids) media containing ammonium sulphate in the concentration range varying from 0–10 mM as sole nitrogen source. The nitrogen starved cells were obtained by incubating the cells from mid-log phase in the SLAD media without the addition of nitrogen source for 8 hrs and thereafter harvested and washed to inoculate into the SLAD media containing ammonium sulphate. The starting inoculum size of OD 1.0 at 600 nm was maintained in all the steady state experiments.

### Ammonium sulphate measurement (Micromolar range)

Ammonium sulphate concentration was determined spectrophotometrically (0.0–200 µM) using o-phthaldialdehyde assay [52].

### Fed batch operation

In order to maintain a steady state concentration of ammonium sulphate, the experiments were carried out in a fed batch mode. The ammonium sulphate concentration was maintained by continuous addition of standard ammonium sulphate solution (100 mM). Different average concentrations (±10%) were maintained by altering the feed rates. Care was also taken that the glucose level remained high (2%) throughout the incubation.

### β-galactosidase assay

Assays were performed with the extracts of culture grown on liquid media. Cells were harvested and disrupted by glass beads and β-galactosidase activity was measured in the cell extract as described by Adams *et al*. (1997)[53]. Assays were carried out in triplicates for each sample. Steady state experiments were repeated at least three times with different transformant each time. Specific activities are represented as nanomoles of product formed per minute per unit OD biomass. The measurement of β-galactosidase from the plasmid-based reporter permitted the reproducible measurement of reporter activity.

### Trehalose measurement

Extracts of culture grown on liquid media were disrupted after exposure to microwave irradiation for 60 sec, which also destroyed the trehalase activity. Trehalose was extracted from the microwave-treated yeast cells using water as solvent for 10 min at room temperature [54]. The extracted trehalose concentration was measured using HPLC (Biorad Aminex column).

### Simulations

Steady state model of the integrative network was developed based on the signaling network existing from the current understanding to quantify the expression of *FLO11* with respect to availability of the nitrogen source. Our previous model of the network comprising of cAMP-PKA and MAPK pathways with respect to the expression of *FLO11* had shown that cAMP-PKA pathway was more sensitive than MAPK pathway [16]. Further, we had demonstrated that the activation level of cAMP-PKA pathway primarily controls the expression of *FLO11*. We were also able to characterize the network behavior as a multiplicative product of two functionalities involving adenylate cyclase, adc and Cdc42p of cAMP-PKA and MAPK pathways, respectively.
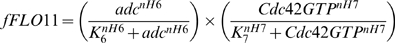
(3)The exponents nH6 = 4 and nH7 = 0.8 represented the Hills coefficient for the fractional expression of *FLO11* (*fFLO11*) with respect to concentration of adenylate cyclase (adc) and Cdc42GTP, respectively. K_6_ and K_7_ are the respective half saturation constants for the fractional activation of cAMP-PKA and MAPK pathways. It can be noted that expression of *FLO11* was more sensitive to cAMP-PKA pathway than MAPK pathway. Here, we included the TOR cascade and Mep2 regulation of cAMP-PKA and MAPK pathways to quantify the network behavior with respect to the availability of nitrogen in the medium. Mep2 is shown to be primarily involved in ammonium transport and in signaling nitrogen availability for filamentous growth. Further, Mep2 function upstream of Gpa2, but the mechanism of activation of Gpa2 by Mep2 is unknown [13]. Genetic studies have shown that the repression of Gpa2 by kelch repeat protein,Gpb1/2 is relieved by Gpr1 with glucose re-addition to glucose starved cells [22]–[26]. Gpr1 is also required for filamentous growth, but no direct interaction with nitrogen source is shown [55]. Therefore, we assumed that Mep2 can activate Gpa2 either through direct downregulation of Gpb1/2 or through Gpr1. Under such a condition, Mep2 can also activate Ras2 by downregulating the Gpb1/2, a known stabilizer of Ira1, thereby controlling MAPK activation. Mep2 as an upstream activator of cAMP-PKA and MAPK pathways is shown in Figure S1. Further, Mep2 expression was not significantly different in high and low concentration of ammonium sulphate [13]. Therefore, regulation based on protein level does not explain its role in pseudohyphal differentiation. Further, Mep2 is actively involved in ammonium transport even under the concentration which repressed filamentous growth indicating that the transport of ammonium was not sufficient for the filamentous growth [13]. Therefore, Mep2 should sense and signal to downstream while transporting under inducing condition. Such a regulation involving transport coupled with signaling can be described by Equation 4.
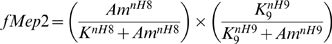
(4)


*fMep2* represented the fractional activation of Mep2 towards filamentous growth, which depended on the concentration of ammonium sulphate (represented as ‘Am’). The first term in Equation 4 represented the activation of Mep2 through ammonium transport, which depended on the K_m_ for ammonium (K = K_m_). The second term represented the inhibition of signaling function of Mep2 at higher concentration of ammonium sulphate. Intracellular glutamine also played a role in regulating the cascades involved in filamentous growth. TOR pathway functions as a glutamine sensor and is shown to have a positive effect in filamentous growth. Further, we assumed that the fractional activation of Tor as a direct function of ammonium sulphate concentration instead of considering the intermediate steps involved in the conversion of ammonium sulphate to glutamine. Thus, the fractional activation of Tor, *fTOR* is given by,
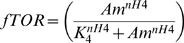
(5)


TOR pathway is shown to function in parallel to cAMP-PKA and MAPK pathways while regulating filamentous growth [9]. TOR and cAMP-PKA pathways are also known to parallely regulate the STRE genes [45], [56]. cAMP-PKA pathway antagonized Msn2/4 dependent transcription of STRE genes by controlling the import/export rate of Msn2/4 translocation. cAMP-PKA pathway reduced the import rate by phosphorylating the nuclear localization signal (NLS) present in Msn2/4 and also increased the export rate during glucose re-addition to glucose starved cells [45]. Interestingly, nitrogen starvation or rapamycin treatment resulted in nuclear accumulation of Msn2/4 without perturbing the activity of cAMP-PKA because Msn2-NLS remained phosphorylated under these conditions [45]. This indicated that TOR pathway controlled Msn2/4 export without disturbing the cAMP-PKA activity and loss of TOR activity was sufficient for Msn2/4 accumulation in the nucleus (Figure S2). Tor kinase exercised this control by negatively regulating the phosphatase, Pph21/22 which was required for decreasing the export rate. Pph21/22 along with its catalytic subunits, Cdc55 and Tpd3 is shown to control the export rate of Msn2/4 [34]. The steady state equation for nuclear translocation of Msn2/4 is given by.
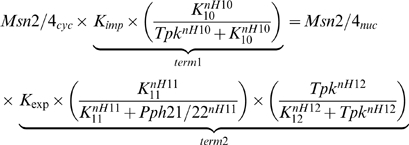
(6)Term1, in Equation 6, represented the regulation of import rate, K_imp_ by PKA of cAMP pathway (given by Tpk) and term 2 represented the regulation of export rate, K_exp_ by phosphatase Pph21/22 of TOR pathway and PKA of cAMP pathway. The negative regulation of phosphatase Pph21/22 by Tor kinase is given by Equation 7.
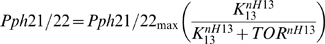
(7)Further, TOR and cAMP-PKA pathways also participated in the synthesis of G1 cyclins, which have a transcriptional role in the regulation of *FLO11* [37], [57]. TOR pathway is shown to control the translation initiation and G1 progression [37], [40]. Loss of Tor activity either through rapamycin treatment or nitrogen starvation is shown to downregulate G1 cyclins synthesis through loss of translational initiation of cyclin Cln3 and is represented by Equation 8 (Figure S2).
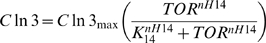
(8)Hyperactivating cAMP-PKA pathway did not prevent the loss of G1 cyclins due to nitrogen starvation suggesting that cAMP-PKA does not control G1 cyclin synthesis under this condition [58]. Furthermore, mitotic cyclin Clb2 is shown to decrease the SBF activation of Cln1/2 expression during mitosis (Figure S2). In our current analysis, we have studied the effect of Tor activity on the expression of *FLO11* mediated through G1 cyclin synthesis and Msn2/4 translocation with respect to the nutritional availability. The steady state model equations and parameters relevant to cAMP-PKA and MAPK pathways were taken from our previous work. The network was modeled at steady state based on the framework reported by Goldbeter and Khoshland [59]. Steady state equations for covalent modification cycles, equilibrium relationship for allosteric interactions and mass balance equations for total species are listed in the supporting information (Text S1). These equations were solved numerically using Fsolve program of MATLAB (The Math-Works Inc.). Most of the parameters for G1 cyclin synthesis and Msn2/4 translocation were taken from the literature and are given as part of supporting information (Text S1). Values for the unknown parameters were evaluated through parametric sensitivity studies to yield biologically relevant responses.

## Supporting Information

Figure S1 Schematic representation of cAMP and MAPK pathways
(0.07 MB PDF)

Figure S2 Schematic representation for TOR mediated control of G1 cyclins and Msn2/4 nuclear translocation
(0.04 MB PDF)

Figure S3 Multiple roles of Tor in control of vegetative growth, filamentous growth and starvation response.
(0.02 MB PDF)

Figure S4 General Methodology to study the steady state behavior of signaling network
(0.06 MB PDF)

Text S1 Steady state equations and relevant parameters for cAMP-PKA, MAPK and TOR pathways.
(0.20 MB PDF)
