# Supplementary material for: Integration of Global Signaling Pathways, cAMP-PKA, MAPK and TOR in the Regulation of FLO11
Source: PLoS One. 2008 Feb 27;3(2):e1663. doi: 10.1371/journal.pone.0001663 (PMC2246015; doi:10.1371/journal.pone.0001663)

Figure S1 *Schematic representation of cAMP and MAPK pathways*. The network was characterized into four different modules. *Module 1*: Upstream regulation of Mep2 by ammonium sulphate concentration. *Module 2*: Ras2 activation involving Cdc25 and Ira1, where Ira1 is regulated by Mep2 and Cdc25 by Kss1 of MAPK cascade. *Module 3*: upstream regulation of Gpa2 by Mep2, which involves removal of Gpb1/2 inhibition *Module 4:* A network comprising of cAMP and MAPK pathways

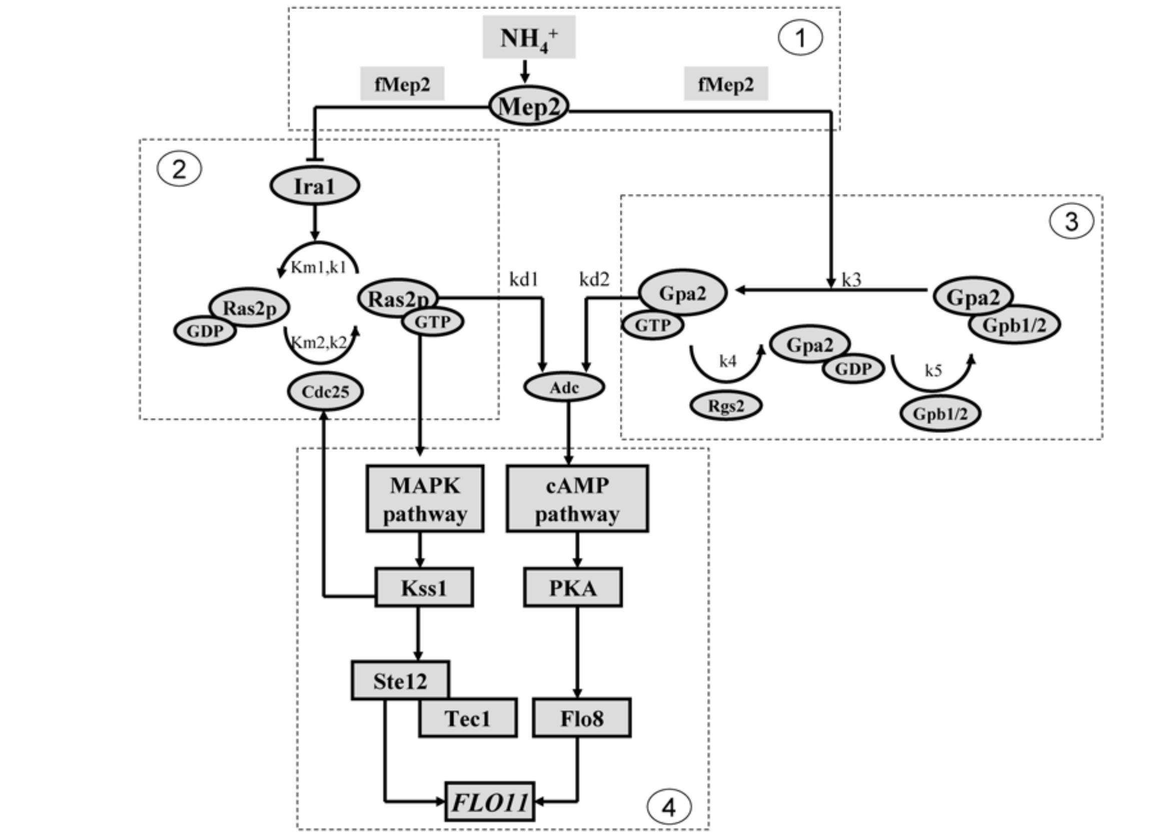

Supplement: Figure S1 — Schematic representation of cAMP and MAPK pathways (0.07 MB PDF) [file pone.0001663.s001.pdf]
