# Supplementary material for: Integration of Global Signaling Pathways, cAMP-PKA, MAPK and TOR in the Regulation of FLO11
Source: PLoS One. 2008 Feb 27;3(2):e1663. doi: 10.1371/journal.pone.0001663 (PMC2246015; doi:10.1371/journal.pone.0001663)

Figure S2 *Schematic representation for TOR mediated control of G1 cyclins and Msn2/4 nuclear translocation.* Tor controls the synthesis of G1 cyclin by controlling the translational initiation of *CLN3* mRNA. Cln3 activates SBF, a transcriptional activator of Cln1/2. SBF is inactivated by mitotic cyclin, Clb2. Tor negatively regulates the phosphatase Pph21/22 under nitrogen rich condition, which is required to decrease the export rate of Msn2/4 from the nucleus. The term Tpk represents the input from cAMP pathway, which is required to decrease the import rate and increase the export rate.

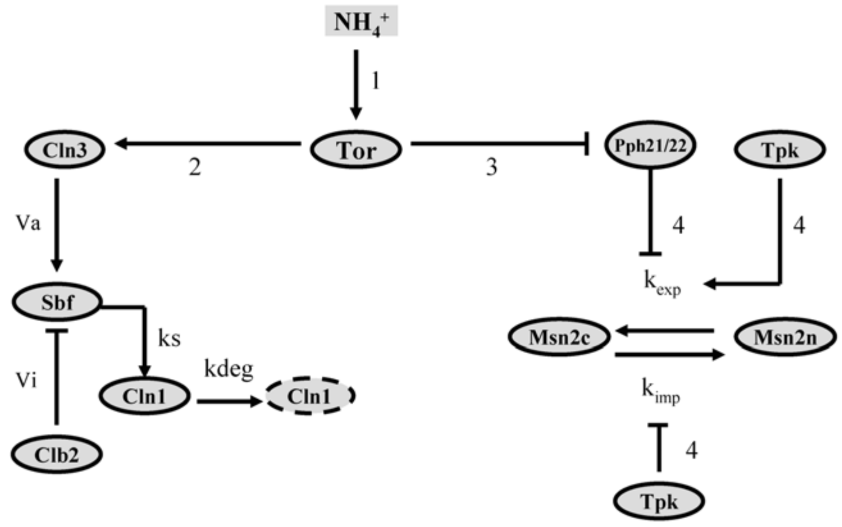

Supplement: Figure S2 — Schematic representation for TOR mediated control of G1 cyclins and Msn2/4 nuclear translocation (0.04 MB PDF) [file pone.0001663.s002.pdf]
