# Supplementary material for: Integration of Global Signaling Pathways, cAMP-PKA, MAPK and TOR in the Regulation of FLO11
Source: PLoS One. 2008 Feb 27;3(2):e1663. doi: 10.1371/journal.pone.0001663 (PMC2246015; doi:10.1371/journal.pone.0001663)

Figure S3 *Multiple roles of Tor in control of vegetative growth, filamentous growth and starvation response.* Tor controls vegetative growth through a subsensitive response, filamentous growth through a bistable response and STRE (starvation) response by remaining switched off (solid line). Tor switches between these states depending upon the availability of nitrogen source. Dotted line indicates the case without negative feedback on Tor activity.

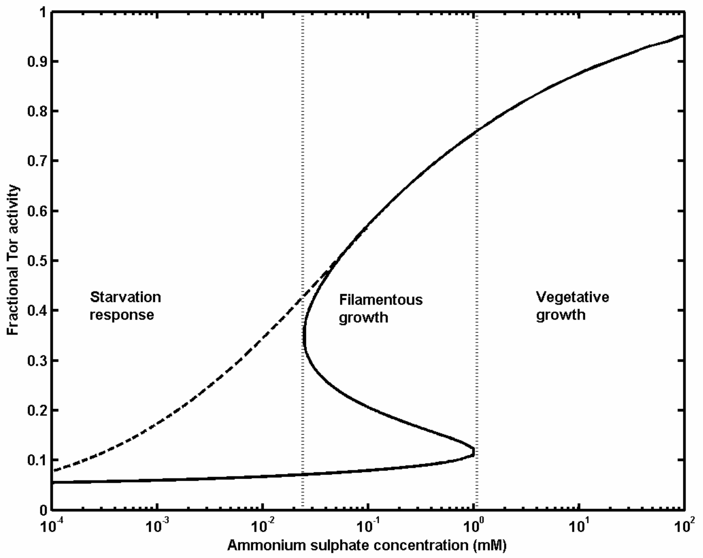

Supplement: Figure S3 — Multiple roles of Tor in control of vegetative growth, filamentous growth and starvation response. (0.02 MB PDF) [file pone.0001663.s003.pdf]
