# Supplementary material for: Integration of Global Signaling Pathways, cAMP-PKA, MAPK and TOR in the Regulation of FLO11
Source: PLoS One. 2008 Feb 27;3(2):e1663. doi: 10.1371/journal.pone.0001663 (PMC2246015; doi:10.1371/journal.pone.0001663)

Figure S4 *General Methodology to study the steady state behavior of signaling network.* The first step involves experimental quantification of phenotypic response with respect to an input at steady state. The observed systemic behavior is analyzed using steady model of the signaling network involved in regulating the phenotype. All the relevant details of the network were collected from the literature and the network was modeled at steady state based on the framework reported by Goldbeter and Khoshland. The unknown parameters were evaluated using parametric sensitivity analysis. The obtained input-output dose response is compared with experimental observation. Based on the comparison results, possible hypothesis can be generated to rationalize the experimental results. The input-output dose response curve, hence obtained reveal the operation and design of the signaling structure that is necessary to elicit the observed systemic properties.

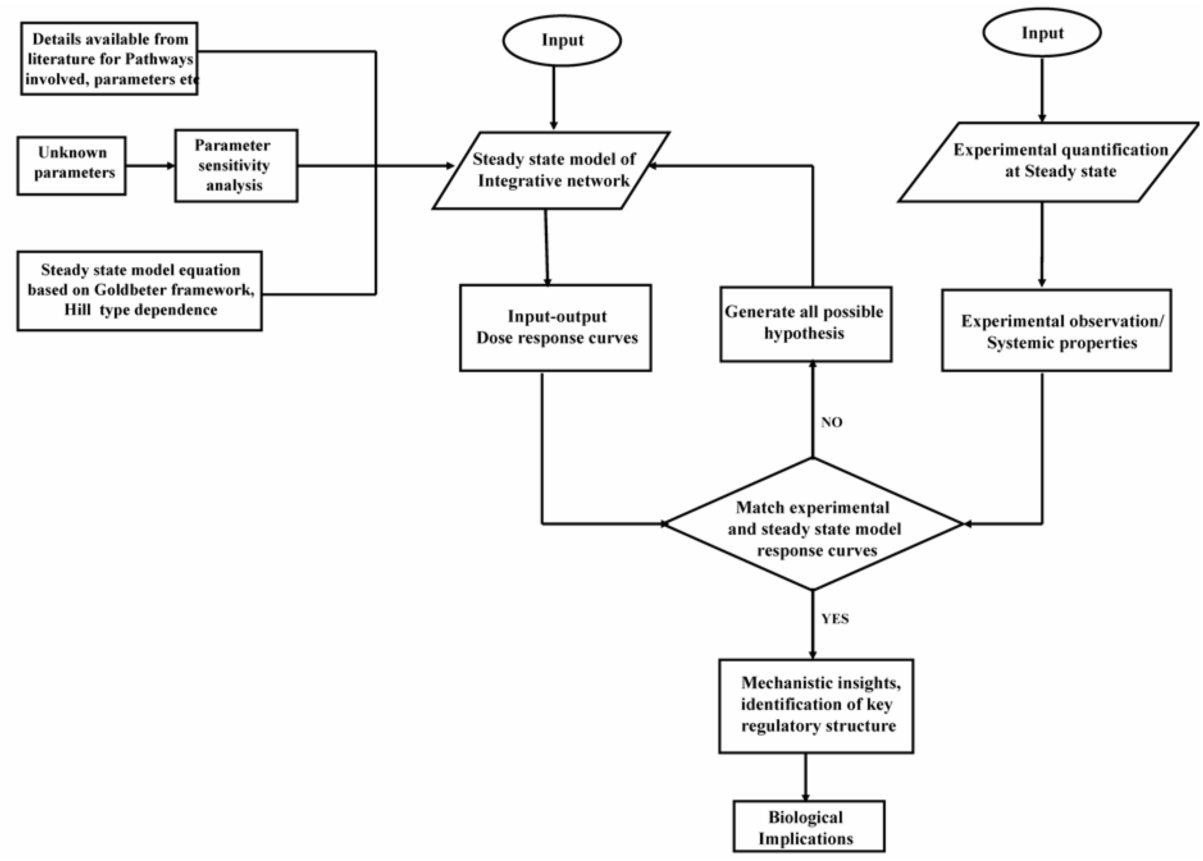

Supplement: Figure S4 — General Methodology to study the steady state behavior of signaling network (0.06 MB PDF) [file pone.0001663.s004.pdf]
