# Supplementary material for: Integration of Global Signaling Pathways, cAMP-PKA, MAPK and TOR in the Regulation of FLO11
Source: PLoS One. 2008 Feb 27;3(2):e1663. doi: 10.1371/journal.pone.0001663 (PMC2246015; doi:10.1371/journal.pone.0001663)
Supplement: Text S1 — Steady state equations and relevant parameters for cAMP-PKA, MAPK and TOR pathways. (0.20 MB PDF) [file pone.0001663.s005.pdf]

Text S1: *Steady state equations and relevant parameters for cAMP-PKA, MAPK and TOR pathways.*

Steady state equations for upstream regulation of cAMP and MAPK pathways by Mep2.

(1) *Fractional activation of Mep2 by ammonium sulphate* ***(Figure S1, Module1)***

$$fMep2 = \left(\frac{Am^{nH8}}{K^{nH8} + Am^{nH8}} \right) \times \left(\frac{K_9^{\;nH9}}{K_9^{\;nH9} + Am^{nH9}} \right)$$

(2) *Ras2 activation module involving cdc25p and Ira1* ***(Figure S1, Module 2)***

$$\frac{k1 \times Ras2GTP \times Ira1}{Km1} = \frac{k2 \times Ras2GDP \times Cdc25}{Km2}$$

Activated Mep2 function to destabilize Ira1 by inactivating Gpb1/2, thereby reduces the active Ira1 concentration. Here, we assume that the total concentration of Ira1 is affected with activation of Mep2, which is given by Ira1t = fMep2 x Ira1max.

(3) *Gpa2 regulation by Mep2* ***(Figure S1, Module 3)***

$$\boldsymbol{k}3 \times \boldsymbol{G\alpha\beta\gamma} \times \boldsymbol{RL} = \boldsymbol{k}4 \times \boldsymbol{G\alpha GTP} \times \boldsymbol{Rgs}2$$

$$\boldsymbol{k}4 \times \boldsymbol{G\alpha GTP} \times \boldsymbol{Rgs}2 = \boldsymbol{k}5 \times \boldsymbol{G\alpha GDP} \times \boldsymbol{G\beta\gamma}$$

These equations represent the regulation of GPCR by binding of ligand to the receptor. Here, Gαβγ represents Gpa2-Gpb1/2 complex, GαGTP represents Gpa2GTP, Gβγ represents Gpb1/2 and GαGDP represents Gpa2GDP. RL represents the receptor-ligand complex. Here, RL represents the Mep2 activated by ammonium sulphate.

Steady state equations relevant to cAMP and MAPK pathways (Figure S1, Module 4) were taken from our previous work [1].

Reaction description	***Equation***
Flo8 activation by Tpk2	$\frac{k11}{Km11}[Flo8][Tpk2] = \frac{k22}{Km22}[Flo8p][E2]$

Pde1p activation

$$\frac{k13}{Km13}[Pde1][Tpk2]=\frac{k14}{Km14}[Pde1p][E14]$$

cAMP rate balance

$$k16[Adc\_a] =\frac{k14}{Km14}[Pde2][cAMP]+\frac{k15}{Km15}[Pde1p][cAMP]$$

Cdc25 activation

$$\frac{k24}{Km24}[Cdc25][Kss1p]=\frac{k25}{Km25}[Cdc25p][E25]$$

Dig1/2 inactivation

$$\frac{k26}{Km26}[Dig1/2][Kss1p]=\frac{k27}{Km27}[Dig1/2p][E27]$$

Ste12Tec1 activation

$$\frac{k28}{Km28}[Ste12Tec1][Kss1p]=\frac{k29}{Km29}[Ste12pTec1p][E29]$$

Ste12Tec1_Dig1/2_kss1 complex phosphorylation by Ste7pp (MAPK activation by MAPKK)

$$\frac{k31}{Km31}[Ste12Tec1\_Dig1/2\_Kss1][Ste7pp] =\frac{k32}{Km32}[Ste12Tec1\_Dig1/2\_Kss1p][E32]$$

Ste7 (MAPKK) activation by Ste20 (MAPKKK)

$$\frac{k34}{Km34}[Ste11p\_Ste50][Ste7p]=\frac{k35}{Km35}[Ste7pp][E35]$$

Ste7p (MPKK) activation by Ste20 (MAPKKK)

$$\frac{k36}{Km36}[Ste11p\_Ste50][Ste7]=\frac{k37}{Km37}[Ste7p][E37]$$

Ste11 (MAPKKK) activation by Ste20 complex (MAPKKKK)

$$\frac{k38}{Km38}[Ste20\_Cdc42GTP\_Bmh1/2][Ste11\_Ste50] =\frac{k39}{Km39}[Ste11p\_Ste50][E39]$$

Cdc42 activation by Cdc24p

$$\frac{k43}{Km43}[Cdc42GDP][Cdc24p] =\frac{k44}{Km44}[Cdc42GTP][Rga1]$$

- Nomenclature: If a protein is 'A' and another protein is 'B' then 'A_B' represents the complex between 'A' and 'B'. Also, 'Ap' represents the activated protein (phosphorylated). Ei represents a phosphatase.
- Relevant parameters (**Rate constants, Michaelis Menten constant, total concentrations and disassociation constants**) for the module cAMP and MAPK (Module 4) were taken from Sengupta et al 2007 [1].

Steady state equations for TOR mediated control of G1 cyclins and Msn2/4 translocation (Figure S2)

(1) *Fractional activation of TOR by ammonium sulfate*

$$fTOR = \left(\frac{Am^{nH4}}{K_4^{nH4} + Am^{nH4}} \right)$$

(2) *Translational control of Cln3 by TOR*

$$C\ln 3 = C\ln 3_{\max} \left(\frac{TOR^{nH14}}{K_{14}^{nH14} + TOR^{nH14}} \right)$$

(3) Inactivation of Phosphatase Pph21/22 by TOR

$$Pph21/22 = Pph21/22_{\max} \left(\frac{K_{13}^{nH13}}{K_{13}^{nH13} + TOR^{nH13}} \right)$$

(4) *Control of nuclear translocation of Msn2/4 by Pph21/22 and Tpk*

$$Msn2/4_{cyc} \times \underbrace{K_{imp} \times \left(\frac{K_{10}^{nH10}}{Tpk^{nH10} + K_{10}^{nH10}} \right)}_{term1} = Msn2/4_{nuc} \times \underbrace{K_{\exp} \times \left(\frac{K_{11}^{nH11}}{K_{11}^{nH11} + Pph21/22^{nH11}} \right) \times \left(\frac{Tpk^{nH12}}{K_{12}^{nH12} + Tpk^{nH12}} \right)}_{term2}$$

(5) *Sbf activation and inactivation by Cln3 and Clb2, respectively*

$$\boldsymbol{Va} = \boldsymbol{kasbf} \times \boldsymbol{C}\ln 3$$

$$\boldsymbol{Vi} = \boldsymbol{k_{isbf}} + \boldsymbol{ki} \times \boldsymbol{Clb}2$$

$$\boldsymbol{kasbf} \times \boldsymbol{C}\ln 3 - (\boldsymbol{k_{isbf}} + \boldsymbol{ki} \times \boldsymbol{Clb}2) \times \boldsymbol{Sbf} = 0$$

(6) *Sbf mediated synthesis and degradation of G1 cyclin Cln1/2*

$$\boldsymbol{ks} \times \boldsymbol{Sbf} - \boldsymbol{k}\deg \times \boldsymbol{C}\ln 1 = 0$$

Species		*Total molar balances*
Ras2t	=	*Ras2GTP + Ras2GDP + Ras2GDP_Cdc25p + Ras2GTP_Ira1*
Irat	=	*Ira1 + (Ras2GTP)(Ira1) /Km1*
Gαt	=	*Gαβγ + GαGTP + GαGDP+ Adc_GαGTP*
Gβγt	=	*Gβγ + Gαβγ*
Msn2t	=	*Msn2nuc + Msn2cyc*
Adct	=	*Adc + Adc_GαGTP + Adc_Ras2GTP*
Flo8t	=	*Flo8 + Flo8p*
E2t	=	*E2 + Flo8p_E2*
Pde1t	=	*Pde1 + Pde1p-Camp + Pde1p + Pde1p_E12 + Pde1_C*
R_2C_2t	=	$2.R_2C_2 + 2\ R_2(Camp)_4$
Pde2t	=	*Pde2 = Pde2t/(1+ [Camp]/Km(2))*
Ct	=	$2\ R_2C_2 + C + Pde1\_C$
Ste12tec_t	=	*Kss1_Dig12_Ste12Tec1 + Kss1p_Dig12_Ste12Tec1 + Ste12Tec1 + Ste12Tec1p + Ste12Tec1_Kss1p + Ste12pTec1p_E29*
Kss1t	=	*Kss1p_Dig12_Ste12Tec1 + Kss1p + Kss1_Dig12_Ste12Tec1 + Kss1 + Ste12Tec1_Kss1p + Kss1p_Dig12_Ste12Tec1 _ E32 + Kss1_Dig12_Ste12Tec1 _Ste7p + Kss1p_ Dig12 + Cdc25_Kss + Kssfus3*
Dig12t	=	*Dig12p + Dig12 + Kss1_Dig12_Ste12Tec1 + Kss1p_Dig12_Ste12Tec1+ Kss1p_Dig12_Ste12Tec1 _ E32 + Kss1_Dig12_Ste12Tec1_Ste7pp + E27_Dig12p + Dig12_ Kss1p*
Cdc25t	=	*Cdc25p + Cdc25 + Cdc25_Kss + Cdc25_Fus3 + Cdc25_Kss1p + Cdc25p_E10*

Fus3t	=	*Fus3 + Cdc25_Fus3 + Kssfus3*
E35t	=	*E35 + (Ste7pp)(E35)/Km(2)*
E37t	=	*E37 + (Ste7p)(E37)/Km(4))*
E39t	=	*E39 + (Ste7p)(E39)/Km(6))*
Ste7t	=	*Ste7 +Ste7pp +Ste7p (intermediate complexes are neglected)*
Ste11ste50t	=	*Ste11ste50 + Ste11pste50*
Ste20t	=	*Ste20_Hl7p + Ste20 + Cdc42p_Ste20_Bmh + Cdc42p_Ste20_Bmh*
Hl7pt	=	*Hl7p + Ste20_Hl7p*
Rga1pt	=	*Rga1p + Cdc42pGTP_Rga1p*
Cdc42pt	=	*Cdc42p_Gtp + Cdc42pGtp_Ste20 + Cdc42p_Ste20_Bmh +Cdc42p_Gdp + Cdc24p_Cdc42pGdp + Rga1p_Cdc42pGtp*

Relevant parameters (Rate constant, Michaelis Menten constant, total concentration and disassociation constant) for TOR pathway and Modules 1, 2 and 3 of Figure S1.

Component Concentrations (nM)

Species	***Conc. (nM)***	***Reference***	***Species***	***Conc.(nM)***	***Reference***
Pph21/22	160*	[4]	Adc	40	[2]
Msn2	2	[4]	Ira	30	[1]
Tor1	15	[4]	Sbf	50	[3]
Cln3	50	[3]	Rgs2	50	[2]
Cln1/2	50	[3]	Gβα	75	[4]
Clb2	50	[3]	Mep2	50	[4]
Gα	75	[4]	Ras2	200	[2]

Unknown concentrations were calculated from molecules numbers obtained from Yeast GFP fusion localization database (http://yeastgfp.ucsf.edu) [4]. Molecule numbers were converted into nM by considering a cell volume of 100fL

* Only 10% of Pph21/22 is actually involved in the TOR signaling [5].

Rate constants

Nomenclature	***Value (min^{-1})***	***Reference***	***Nomenclature***	***Value (min^{-1})***	***Reference***
k1	36	[7]	kasbf	0.38	[3]
k2	12	[7]	ki	8	[3]
k3	0.5	[2]	kisbf	0.8	[3]
k4	4	[2]	ks	0.15	[3]
k5	2.5	[2]	kdeg	0.12	[3]
kimp	1	[assumed]	kexp	1	[assumed]

Dissociation constants			***Michaelis Menten constants***		
Kd	***Value (nM)***	***Reference***	***Km***	***Value (nM)***	***Reference***
kd1	10	[6]	Km1	250	[7]
kd2	2	[2]	Km2	160	[7]

Hills Coefficient

nH	***value***	***Reference***	***nH***	***value***	***Reference***
nH1	4	[1]	nH9	2	[calculated]
nH2	0.8	[1]	nH10	2	[assumed]

nH3	3 - 4	[calculated]	nH11	3 - 4	[calculated]
nH4	0.3	[calculated]	nH12	2	[assumed]
nH5	2- 3	[calculated]	nH13	0.9 - 1.2	[calculated]
nH6	4	[1]	nH14	2	[calculated]
nH7	0.8	[1]			
nH8	1- 2	[calculated]			

K0.5	***Value (nM)***	***Reference***	***K0.5***	***Value(nM)***	***Reference***
K1	67	[1]	K9	1000*	[calculated]
K2	10	[1]	K10	10	[assumed]
K3	2.5	[calculated]	K11	3	[calculated]
K4	15-30*	[calculated]	K12	10	[assumed]
K5	5	[calculated]	K13	2	[calculated]
K6	14	[1]	K14	7.5	[calculated]
K7	2	[1]			
K	2-16 *	[calculated]			

* Units in μM based on the extracellular ammonium sulphate concentration

References

1. Sengupta N, Vinod PK, Venkatesh KV (2007) Crosstalk between cAMP-PKA and MAP kinase pathways is a key regulatory design necessary to regulate *FLO11* expression. Biophys Chem 125:59-71.

2. Bhalla US (2002) Use of Kinetikit and GENESIS for modeling signaling pathways, Methods Enzymol. 345: 3–23.

3.. Chen, KC, Calzone L, Csikasz-Nagy A, Cross FR, Novak B, Tyson JJ (2004) Integrative analysis of cell cycle control in budding yeast. Mol Biol Cell 15:3841-3862.

4. Ghaemmaghami S, Huh WK, Bower K, Howson R W, Belle A, Dephoure N, O'Shea EK, Weissman JS (2003) Global analysis of protein expression in yeast Nature. 425: 737-741.

5. DiComo CJ, Arndt KT (1996) Nutrients, via the Tor proteins, stimulate the association of Tap42 with type 2A phosphatases. Genes & Dev 10: 1904-1916.

6. Shima F, Yuriko YK, Yanagihara C, Tamada M, Tomoyo O, Kariya K, Kataoka T (1997) Effect of association with adenylyl cyclase-associated protein on the interaction of yeast adenylyl cyclase with Ras protein, Mol Cell Biol 17: 1057–1064.

7. Steven AH, Broach JR (1997) Cdc25p, the guanine nucleotide exchange factor for the Ras proteins of *Saccharomyces cerevisiae*, promotes exchange by stabilizing Ras in a nucleotide-free state. J Biol Chem 269: 16541–16548.
